# A sporadic case of unilateral acrokeratoelastoidosis in Saudi Arabia: a case report

**DOI:** 10.1186/1752-1947-8-143

**Published:** 2014-05-08

**Authors:** Hassan S AlKahtani, Ahmed A AlHumidi, Abdullah H Al-Hargan, Ahmed A Al-Sayed

**Affiliations:** 1 Division of Dermatology, Department of Medicine, King Abdulaziz Medical City, Riyadh, Saudi Arabia; 2 Department of Pathology, King Khalid University Hospital, King Saud University, Riyadh, Saudi Arabia; 3 College of Medicine, King Saud University, P.O. BOX 50652, Riyadh 11533, Saudi Arabia

**Keywords:** Acrokeratoelastoidosis, Genodermatosis, Hyperkeratosis

## Abstract

**Introduction:**

Acrokeratoelastoidosis is a rare condition characterized by bilateral multiple hyperkeratotic papules on the palms, soles, and dorsum of the hands and feet. To the best of our knowledge, only around 40 cases of acrokeratoelastoidosis have been reported worldwide, which are mostly familial.

**Case presentation:**

We report the first case from Saudi Arabia in a 5-year-old Saudi girl of Arabian origin, who presented symptoms of acrokeratoelastoidosis with unilateral sporadic keratosis on her left hand and left foot. We also review the clinical and histopathologic features, etiology, differential diagnosis and its treatment.

**Conclusions:**

Given the rarity of acrokeratoelastoidosis, little is known about the disease. Further studies are required to understand the pathogenesis of the disease for better treatment options. Additional case reports of acrokeratoelastoidosis will help in recognizing risk factors, patient characteristics, environmental influences and possibly new etiological factors.

## Introduction

Acrokeratoelastoidosis (AKE) is a rare type of palmoplantar keratosis that manifests as round or oval papules distributed along the marginal borders of the palms and soles. Reported cases are either sporadic or familial where the familial cases are mostly autosomal dominant. Several treatments have been tried with variable success. The most common histopathologic features of AKE are hyperkeratosis, acanthosis and elastorrhexis. Here, we present a case of AKE in a 5-year-old Saudi girl and a review of the literature for the clinical presentation, histopathologic features, etiology, differential diagnosis, and treatment of the disease.

AKE was first described by the Brazilian dermatologist, Oswaldo Costa in 1953 [1,2]. It is one of the rare groups of diseases that are believed to be due to some unknown pathophysiological process where genetic variations or heredity may be one of the main causal factors. AKE was believed to have a racial predilection towards Arabs and Negroes; although this hypothesis has been challenged [3].

For the differential diagnosis of AKE, focal acral hyperkeratosis has a similar clinical appearance, but during histopathological examination, elastorrhexis is not seen and alterations are confined to the epidermis (hyperkeratosis and acanthosis) [3]. Another condition with a similar clinical appearance to AKE is marginal keratoelastoidosis, which is generally associated with intense sun exposure, marked actinic damage, and genetic inheritance. This condition includes: focal acral hyperkeratoses, AKE, papulotranslucent acrokeratoderma, mosaic acral keratosis, and punctuate palmoplantar keratoderma [3]. Other conditions that should be taken into consideration in the differential diagnosis of AKE include acrokeratosis verruciformis of Hopf, degenerative collagenous plaques, and punctate palmoplantar keratoderma [4]. An association with systemic and localized scleroderma was reported by Yoshinaga and Tajima, raising the question of an autoimmune process [5,6]. Awareness of AKE with appropriate clinical examination confirmed by a skin biopsy can facilitate its diagnosis. To the best of our knowledge there is no published report of AKE from Saudi Arabia and, therefore, we believe that this is the first case report from our country.

## Case presentation

**Figure 1:**
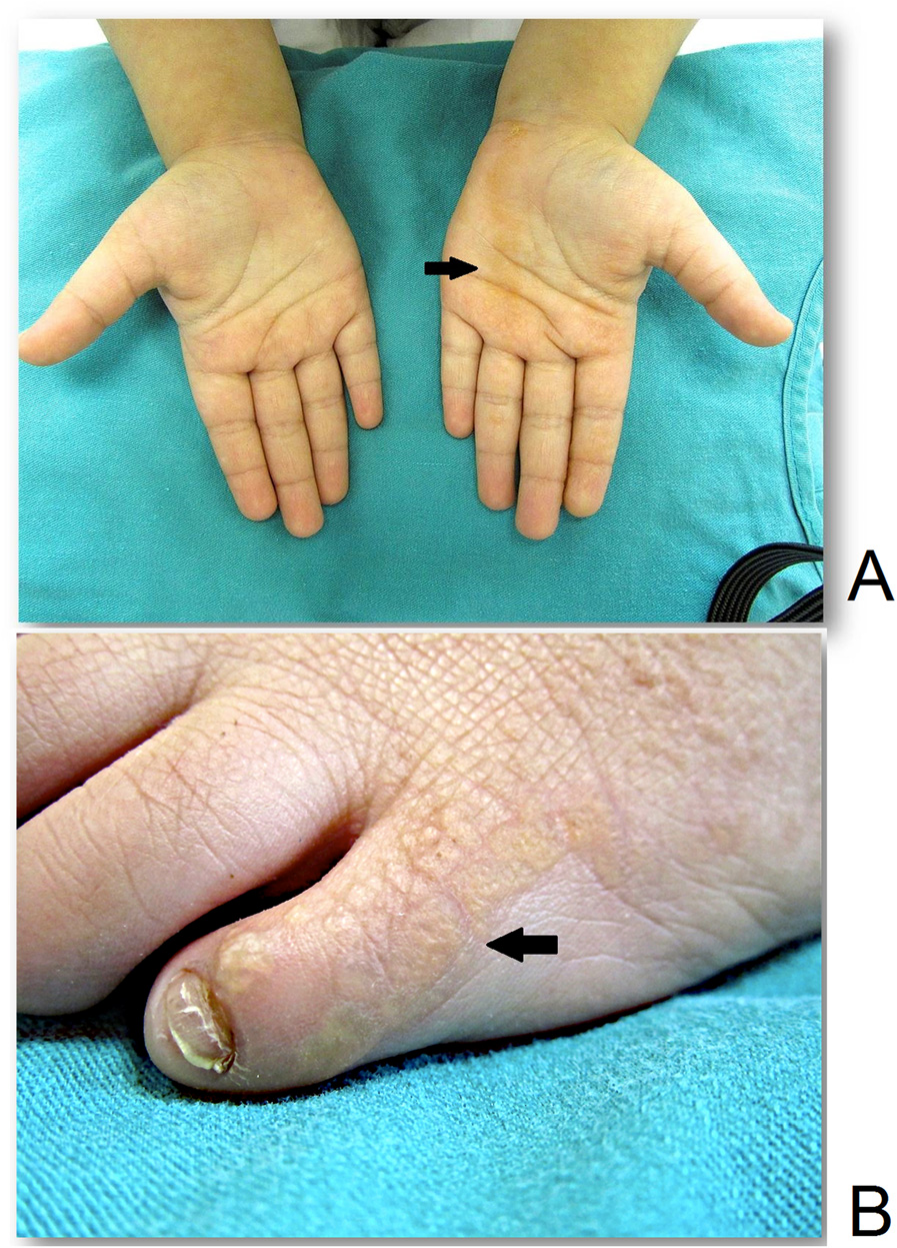
**Gross pathology of acrokeratoelastoidosis. A**: A comparison between the right hand that is free of lesions and the left hand where the skin lesion is distributed in a liner fashion (arrow). **B**: Left lateral margin of the left foot showing multiple yellowish hyperkeratotic papules (arrow).

A 5-year-old Saudi girl of Arabian origin presented at King Abdulaziz Medical City, King Fahad National Guard Hospital’s dermatology clinic with a history of asymptomatic skin lesions on her left hand and left foot (Figure 1). Her parents reported that these lesions had been present since birth, but are spreading and becoming more obvious over the years. The child had a history of hyperhydrosis, which was exclusive to her left hand and left foot. The child had no history of excessive sun exposure or trauma, and no member of her family had similar lesions. Previous treatment consisted of topical calcipotriol without any improvement.

Clinical examination showed multiple yellowish hyperkeratotic papules, coalescing into verrucous plaques that were distributed in a line along the marginal surfaces of her left hand and foot. On her left hand, the lesions were on the palmer surface extending from the thenar eminence to the proximal part of her fourth finger. On her left foot, they were on the lateral side of her foot and fifth toe distally.

**Figure 2:**
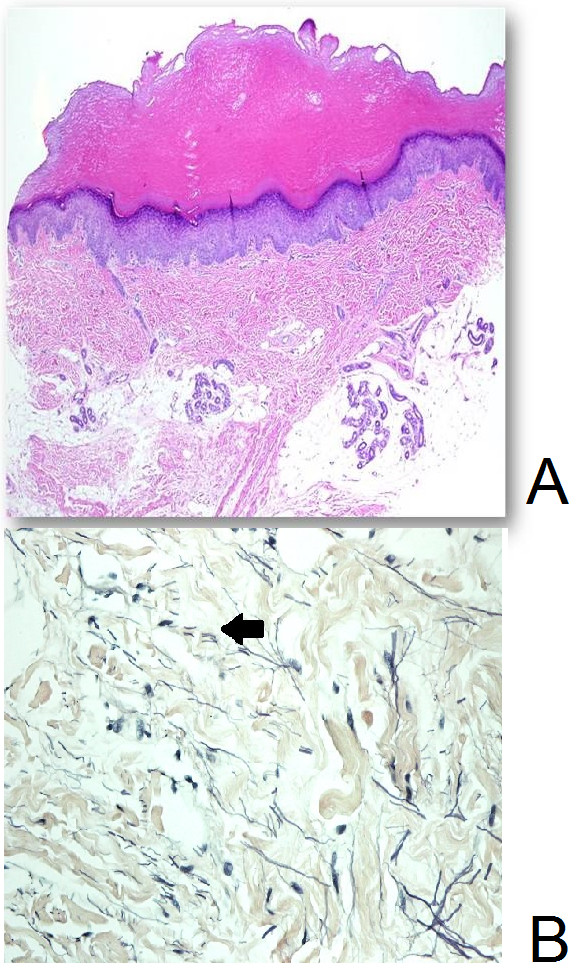
**Histopathology of acrokeratoelastoidosis. A**: Hematoxylin and eosin stain showing hyperkeratosis with acanthosis (hematoxylin and eosin stain, original magnification × 40). **B**: Verhoeff’s-Van Gieson staining showing fragmented thinned elastic fibers (arrow) (original magnification × 400).

Histological examination of a 4mm punch biopsy specimen taken from a papule on her left foot revealed hyperkeratosis and acanthosis (Figure 2A). The special staining for elastic fibers (Verhoeff’s-Van Gieson stain) showed fragmentation and a reduced number of elastic fibers causing significant thinning of the dermis (Figure 2B), confirming the case is AKE. She was given topical calcipotriol for the affected areas on her hand and foot. After 6 months of treatment, the parents reported no significant improvement.

## Discussion

AKE is a rare disease, and while its pathogenesis remains unclear, the onset of the disease is frequently seen in infancy or adolescence [7]. Most cases of AKEs that have been reported so far have occurred before the second to third decades of life. However, the youngest reported patient was 3 years of age [8].

For sporadic forms of the disease, chronic trauma and excessive sun exposure have been proposed as possible causal factors. For familial forms of the disease, the most common inheritance mode is autosomal dominant, most probably related to chromosome 2 [9], but recessive forms have also been reported [10,11]. It is theorized that the keratotic papules that are seen clinically in AKE may be the result of an abnormality in the secretion or excretion of elastic material by fibroblasts in the dermis [11].

The clinical characteristic of AKE is multiple, yellowish papules that are sometimes shiny and keratotic. The papules can be located symmetrically on the lateral surfaces of the palms and soles; the space between the thumb and the forefinger; the anterior surface of the lower legs; and over the knuckles and nail folds [11]. Hyperhydrosis is also frequently observed [12]. The distribution of papules is generally bilateral, but unilateral forms have been reported similar to our patient [6]. However, due to the rarity of the disease, the unilateral variants of AKE are very difficult to identify and it has been suggested that they are due to genetic mosaicism [13].

Typical histopathological findings in AKE include hyperkeratosis with hypergranulosis, acanthosis, and epidermal hyperplasia. Van Gieson stain shows reduced number of elastic fibers in the dermis with a high proportion of fragmented fibers. There is an abnormal deposition or repair of connective tissue within the affected area. Usually, no treatment is required because lesions are asymptomatic with no adverse effects [14], but the condition may be cosmetically unpleasant to patients. Various treatments that are reported include topical and systemic medication, but these are of little or no benefit [15]. Recently, Erbil *et al*. suggested that erbium:YAG laser surgery may be considered a possible therapy. However, patients should be informed of the limited clinical improvement obtained with this treatment [16].

## Conclusions

Given the rarity of AKE, little is known about its incidence, etiology, and pathophysiology. Further studies are needed to address its associations with other diseases including autoimmune diseases. Genetic studies may reveal more about the disease and, therefore, may be helpful in identifying better treatment options. Additional case reports of AKE will help in recognizing the risk factors, patient characteristics, environmental influences, and possibly new etiological factors.

## Consent

Written informed consent was obtained from the patient’s legal guardian for publication of this case report and accompanying images. A copy of the written consent is available for review by the Editor-in-Chief of this journal.

## Abbreviations

AKE: Acrokeratoelastoidosis.

## Competing interests

The authors declare that they have no competing interests.

## Authors’ contributions

HSAlK was the principal author and major contributor in writing the manuscript. AAAlH analyzed and interpreted the patient data. AHAlH reviewed the literature and AAAlS read and corrected the manuscript. All authors read and approved the final manuscript.
